# Case Report: Distal tibial physeal injury secondary to conservative treatment of acute hematogenous osteomyelitis resulting in ankle deformity — highlighting the necessity of regular follow-up

**DOI:** 10.3389/fsurg.2026.1827366

**Published:** 2026-09-30

**Authors:** Tuo Cheng, Jie Li

**Affiliations:** 1 The First Clinical Medical College, Shandong University of Traditional Chinese Medicine, Jinan, Shandong, China; 2 Pediatric Orthopaedics, Affiliated Hospital of Shandong University of Traditional Chinese Medicine, Jinan, Shandong, China

**Keywords:** acute hematogenous osteomyelitis (AHO), ankle deformity, epiphysiodesis, osteotomy, physeal injury

## Abstract

Ankle deformities most commonly result from physeal injuries following major trauma; those secondary to occult physeal injury without significant antecedent trauma are rarely documented. We report a case of ankle deformity in a 13-year-old boy resulting from distal tibial physeal injury following conservative treatment for acute hematogenous osteomyelitis(AHO) four years earlier; despite initial clinical improvement, the patient was subsequently lost to follow-up. At his return visit, significant ankle discomfort severely compromised daily activities. Following combined distal tibial osteotomy and distal fibular epiphysiodesis, the patient regained normal quality of life. This case highlights a frequently overlooked aspect of AHO in children: despite initial symptom resolution with conservative treatment, regular follow-up is essential to detect occult physeal injury and prevent irreversible deformity. Early diagnosis facilitates timely intervention and may obviate the need for osteotomy. In managing ankle deformity secondary to AHO, combined distal tibial osteotomy and epiphysiodesis effectively corrects the deformity while preserving subsequent growth balance, representing a reliable therapeutic option.

## Introduction

Osteomyelitis is an inflammatory process of the bone and marrow induced by pathogenic microorganisms, typically characterized by bone destruction, necrosis, and subsequent new bone formation (1). Clinically, it is classified as either acute or chronic, and based on pathogenesis, further subdivided into hematogenous, contiguous-focus, and traumatic categories (2, 3). Among these, AHO is one of the most prevalent musculoskeletal infections in childhren (4). However, delayed diagnosis or inadequate initial management predisposes to progressive bone destruction, chronic disease course, and persistent functional impairment (5). The primary goal of treatment of the disease is to achieve effective infection control and promote the repair of damaged tissues. Definitive therapy should be guided by antimicrobial susceptibility testing. In selected cases, this medical regimen must be supplemented by thorough debridement of necrotic bone and surrounding soft tissues (6). For uncomplicated AHO in children, early systemic antibiotic therapy constitutes first-line treatment, whereas surgical intervention is typically reserved for cases refractory to medical management or those demonstrating substantial abscess formation on imaging (7, 8).

In this report, we describe a 13-year-old boy presenting in 2025 with ankle deformity secondary to distal tibial physeal injury. Notably, the patient had no history of significant trauma; retrospective review revealed a prior episode of AHO four years earlier. Correlation of imaging findings with the medical history suggests that the physeal destruction is attributable to this prior hematogenous infection. This case highlights that conservative management of AHO in children carries a risk of occult physeal injury, which may progress to premature physeal closure and late-onset joint deformity. Consequently, even amid marked clinical improvement, long-term, protocol-driven iconography surveillance is imperative.

## Case presentation

In 2025, a 13-year-old boy presented to our department with a 1-year history of progressively worsening discomfort in the left ankle. His guardian reported a progressive decline in athletic performance that markedly impaired his daily activities. Physical examination revealed pronounced asymmetrical wear on the lateral sole of the left shoe. A detailed history excluded any recent trauma. Further inquiry revealed a prior episode of AHO of the left distal tibia in 2021, which had been managed conservatively with subsequent clinical remission. However, the patient had failed to adhere to scheduled follow-up protocols. Physical examination revealed a mild antalgic gait and a noticeable varus deformity of the left ankle. The range of motion (ROM) was severely restricted, with plantarflexion(PF) 28°(contralateral: 42°) and dorsiflexion(DF) 4°(contralateral: 12°). Deep tenderness was elicited upon palpation of the medial malleolus. The American Orthopaedic Foot & Ankle Society (AOFAS) Ankle-Hindfoot Scale score was 41 points, indicating a poor functional outcome. The Visual Analogue Scale (VAS) for pain was 5/10 during walking and 2/10 at rest. There were no signs of active infection, including localized erythema, warmth, swelling, or sinus tract formation.

Plain radiographs (Figure 1) demonstrated a tibial articular surface (TAS) angle of 69° on the affected side, indicating a significant varus tilt of the distal tibial joint surface. The distal tibiofibular syndesmosis appeared anatomically preserved without evidence of diastasis or degenerative changes. Computed tomography (CT; Figure 2) revealed extensive central closure of the left distal tibial physis with disorganized trabecular architecture. Mild incongruity of the left ankle joint was noted. The distal tibial medullary canal exhibited heterogeneous density, characterized by multifocal areas of sclerotic bone interspersed with central lucency. Magnetic resonance imaging (MRI; Figure 3) confirmed central physeal closure. The distal tibial marrow signal was heterogeneous, showing focal areas of low T1 and high T2 fat-saturated signal intensity. Similar high-signal foci were observed in the distal fibula and tarsal bones on fat-saturated T2-weighted images. The periarticular soft tissues were unremarkable. Preoperative laboratory evaluations, including complete blood count and C-reactive protein (CRP) levels, were within normal limits.

**Figure 1:**
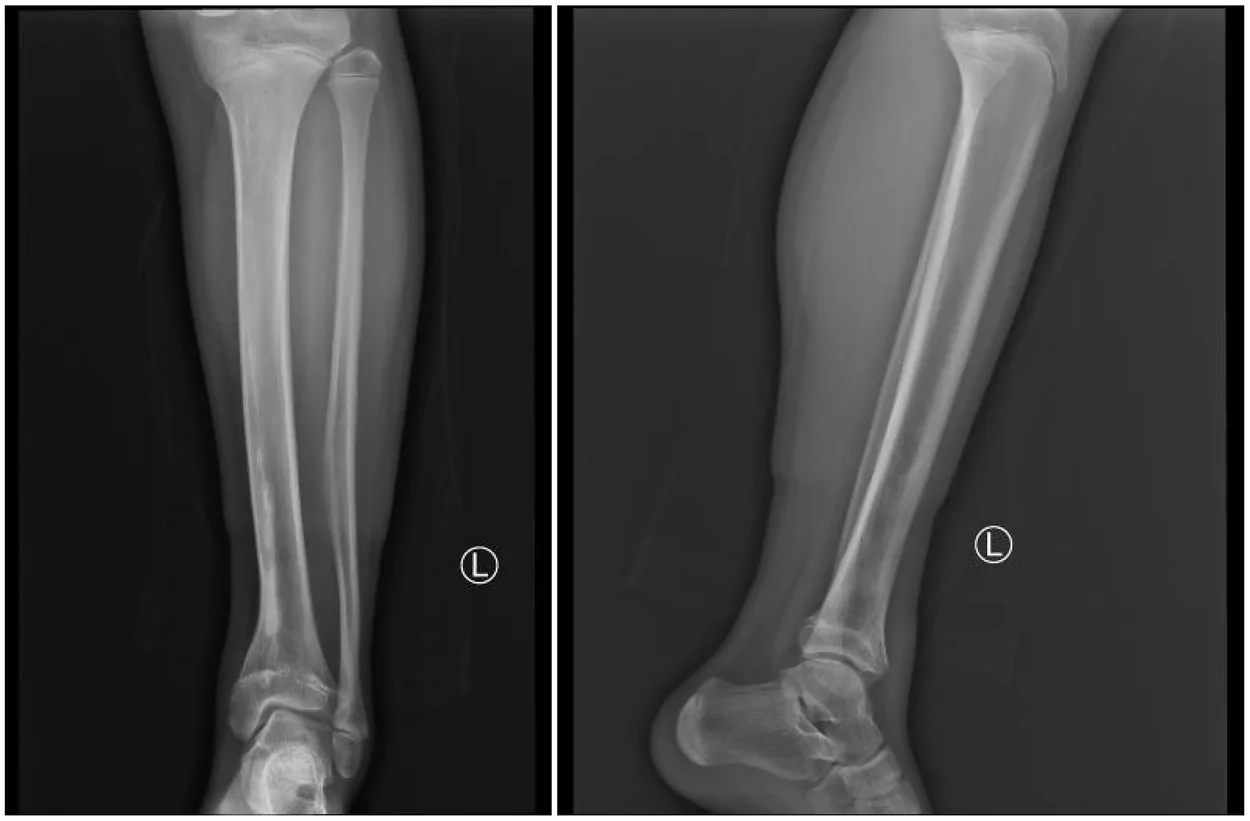
Preoperative plain radiographs of the left lower leg.

**Figure 2:**
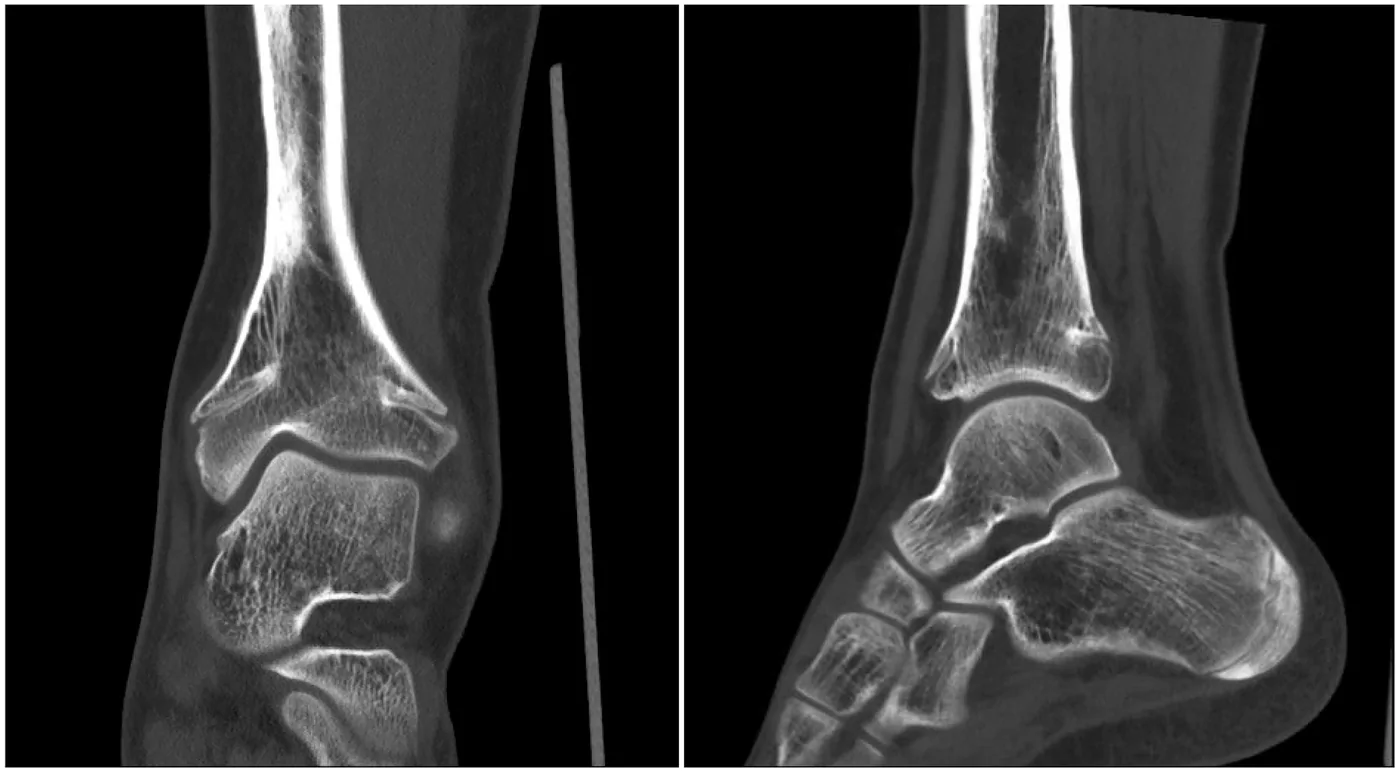
CT images of the left distal tibia and ankle joint.

**Figure 3:**
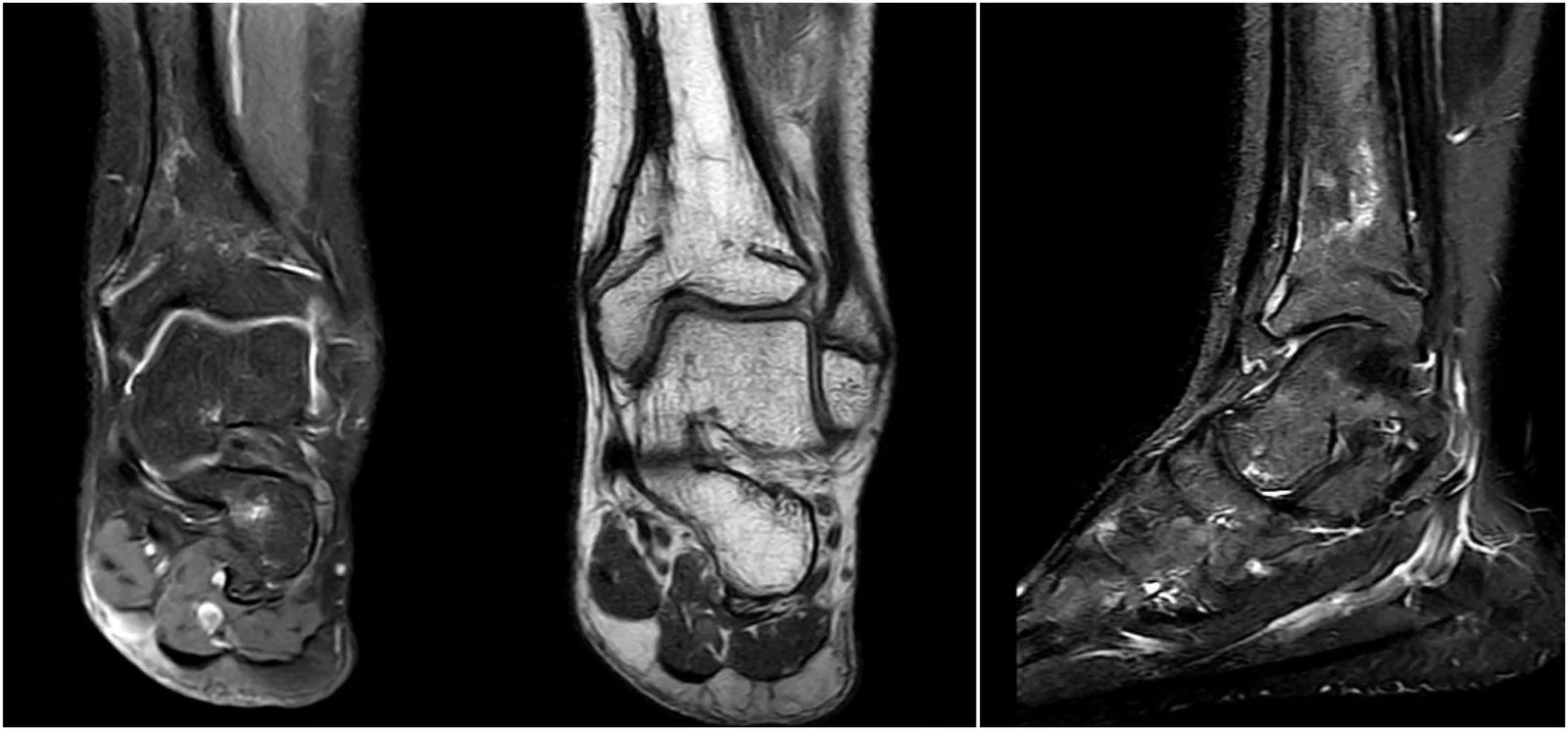
MRI of the left distal tibia and ankle.

Collectively, the clinical history, imaging findings, and laboratory results allowed for a definitive diagnosis. The absence of systemic inflammatory signs, normal serological markers (CRP, WBC), and the lack of imaging features suggestive of active infection—such as abscess formation, diffuse bone marrow edema, or rim enhancement—effectively ruled out a new episode of acute hematogenous osteomyelitis. Similarly, the lack of trauma history and the absence of post-traumatic radiographic features excluded a traumatic etiology for the physeal injury. Instead, the extensive central physeal closure with concomitant bony bridge formation was consistent with a sequela of the prior 2021 AHO. We concluded that the physeal damage resulted from vascular compromise of the distal tibial physis secondary to the previous hematogenous infection, representing a classic post-infectious growth disturbance.

Given that the ankle deformity significantly compromised the patient's daily activities and that the presence of a physeal bony bridge portended progressive worsening, surgical intervention was undertaken following thorough discussion with the patient and his guardians. The surgical strategy aimed to achieve two primary objectives: (1) to perform a distal tibial osteotomy to correct the varus deformity and restore physiological biomechanics and anatomical alignment; and (2) to perform a distal fibular epiphysiodesis to counteract the relative overgrowth of the fibula secondary to tibial growth arrest. This combined approach was designed not only to alleviate current symptoms but also to ensure sustained correction until skeletal maturity, thereby optimizing long-term quality of life and aiming for ankle function comparable to that of healthy peers.

### Surgical technique

The patient was placed in the supine position under general anesthesia. After routine sterile preparation and draping, a 10-cm curvilinear incision was made proximal to the medial malleolus. Dissection proceeded through subcutaneous tissues with careful protection of the saphenous vein and nerve. The fascia was incised, and subperiosteal dissection exposed the medial distal tibial metaphysis and physis. Under C-arm fluoroscopic guidance, a Kirschner wire (K-wire) was inserted parallel to the tibial plafond to mark the proximal extent of the physis, serving as a reference for the osteotomy. The osteotomy was then performed along this wire using an osteotome, deliberately preserving the lateral cortical hinge. A spreader was used to gradually open the medial osteotomy gap, after which the initial guide wire was removed. Intraoperative fluoroscopy confirmed restoration of mechanical alignment, aided by temporarily placing an external reference K-wire perpendicular to the physis (Figure 4A). Fixation was achieved using a dual-plate construct to enhance stability. A posterior-medial T-shaped locking plate and an anteromedial reconstruction plate were contoured to match the native tibial anatomy and secured with locking screws. Final fluoroscopic images confirmed satisfactory limb alignment, symmetric ankle mortise, and optimal hardware positioning. The resultant defect was packed tightly with cancellous allograft bone chips. Subsequently, distal fibular epiphysiodesis was performed under fluoroscopic control. Two guidewires were inserted perpendicular to the distal fibular physis, followed by placement of two 4.0-mm cannulated screws in a crossed configuration across the physis to achieve complete growth arrest (Figures 4B,C). After confirming satisfactory implant positioning and performing a thorough surgical count, the wound was irrigated copiously with saline, and hemostasis was ensured. A penrose drain was placed adjacent to the osteotomy site. The incision was closed in layers, dressed sterilely, and the left ankle was immobilized in a functional brace.

**Figure 4:**
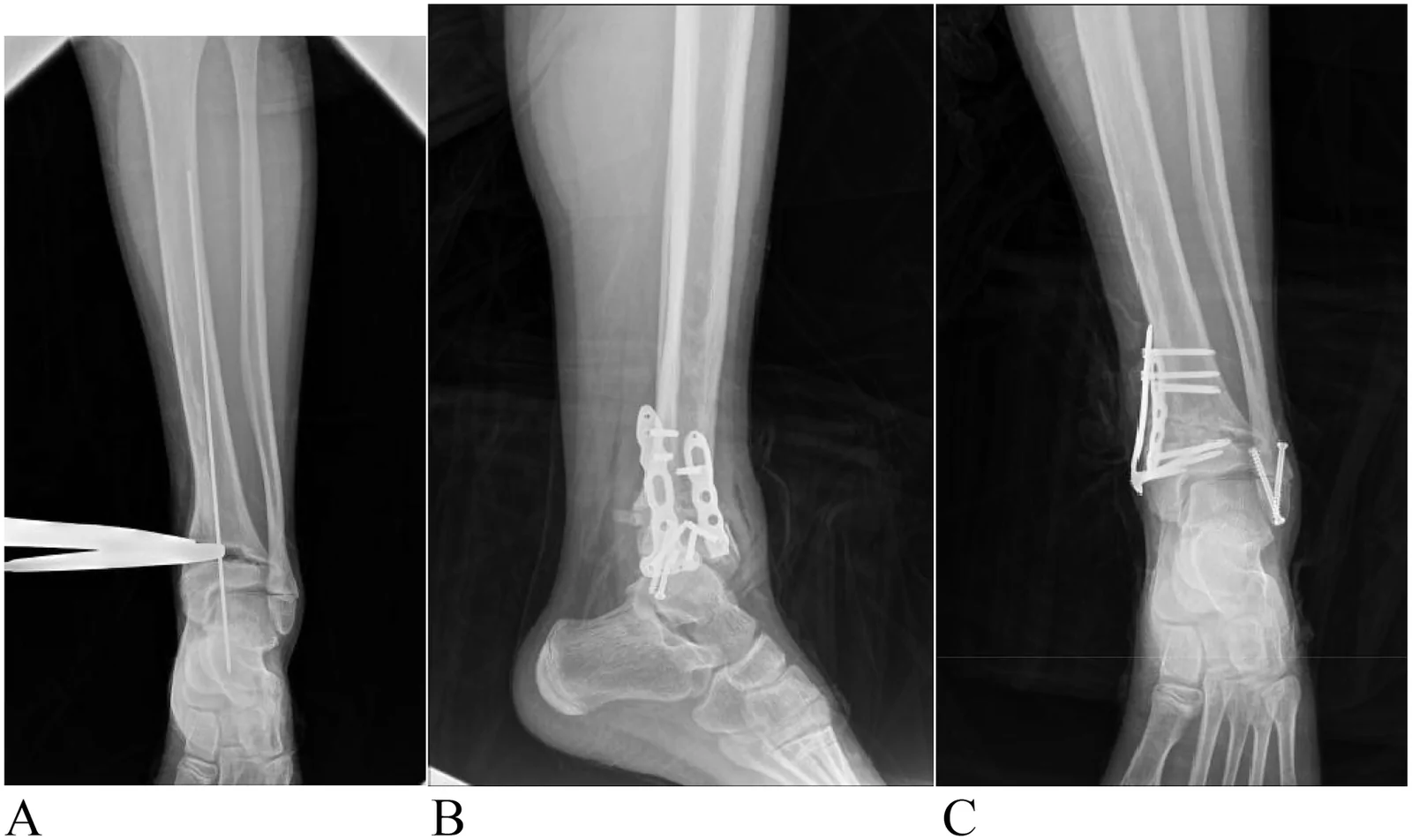
Intraoperative images of the left distal tibia/ankle region. **(A)** shows an X-ray of an ankle with a long thin metal rod serve as an external marker. **(B)** and **(C)** are the anteroposterior and lateral X-ray films after the completion of the surgery.

### Follow-up and outcomes

Postoperatively, serial complete blood counts and CRP levels remained within normal limits. At the 2-week follow-up, the incision had healed primarily with no clinical signs of infection, and the patient was discharged. The patient was scheduled for regular clinical and radiographic evaluations, with serial documentation of AOFAS and VAS scores. Emphasizing gradual restoration of range of motion and progressive weight-bearing as tolerated. The imaging examinations at 4 weeks, 8 weeks and 6 months after operation are shown in Figures 5–7, respectively. At the final follow-up 12 months postoperatively, the patient and his guardian reported that he was able to return to school sports without pain and felt that his ankle function was “almost the same as before the problem started.” Radiographs demonstrated solid bony union at the osteotomy site with maintenance of corrected alignment (Figure 8). Given the patient's age, skeletal maturity, elective hardware removal was performed. The procedure was uneventful, with primary wound healing and excellent functional recovery. Table 1 summarizes the clinical and radiological progress throughout the follow-up period.

**Figure 5:**
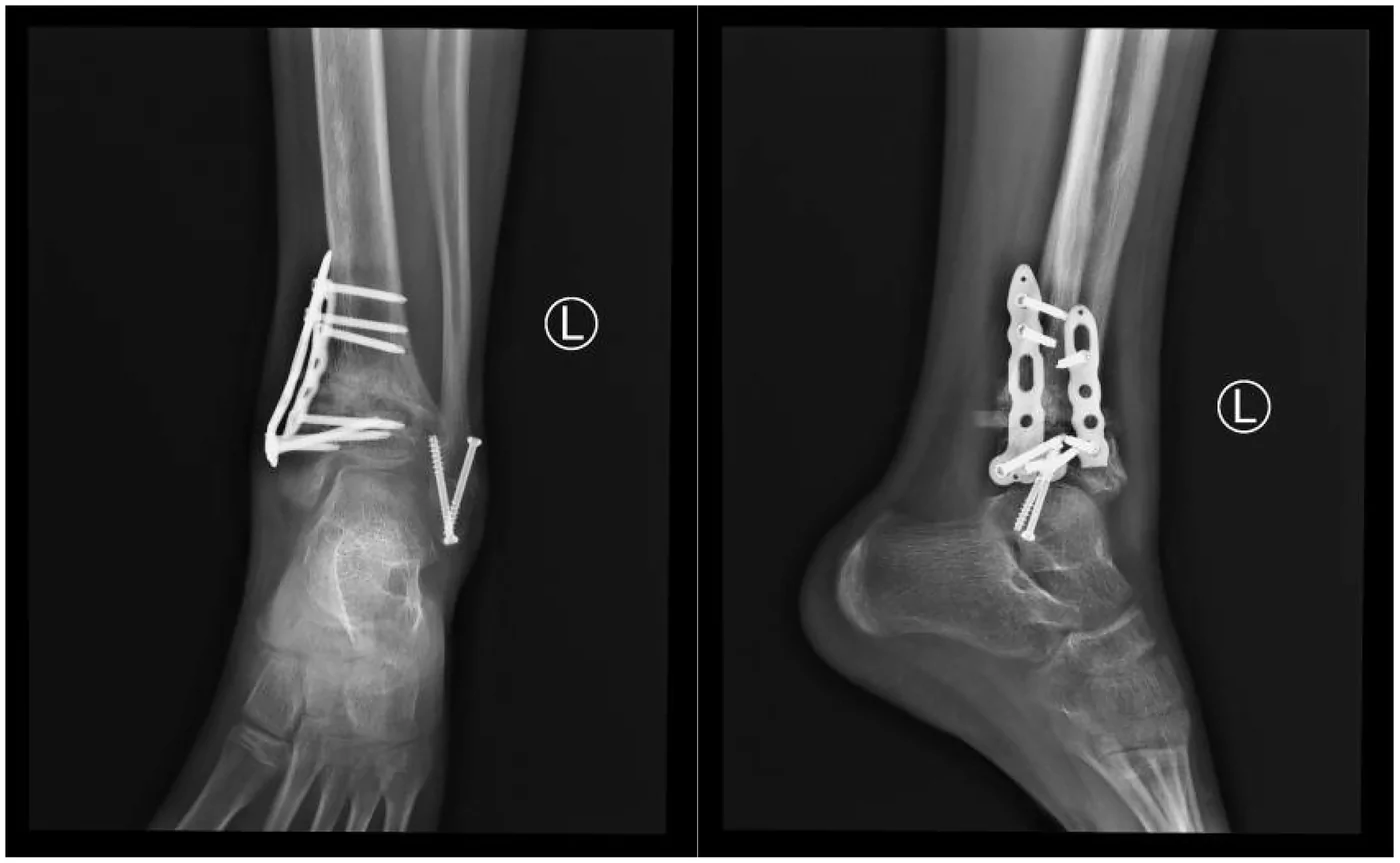
Postoperative radiographs at 4 weeks.

**Figure 6:**
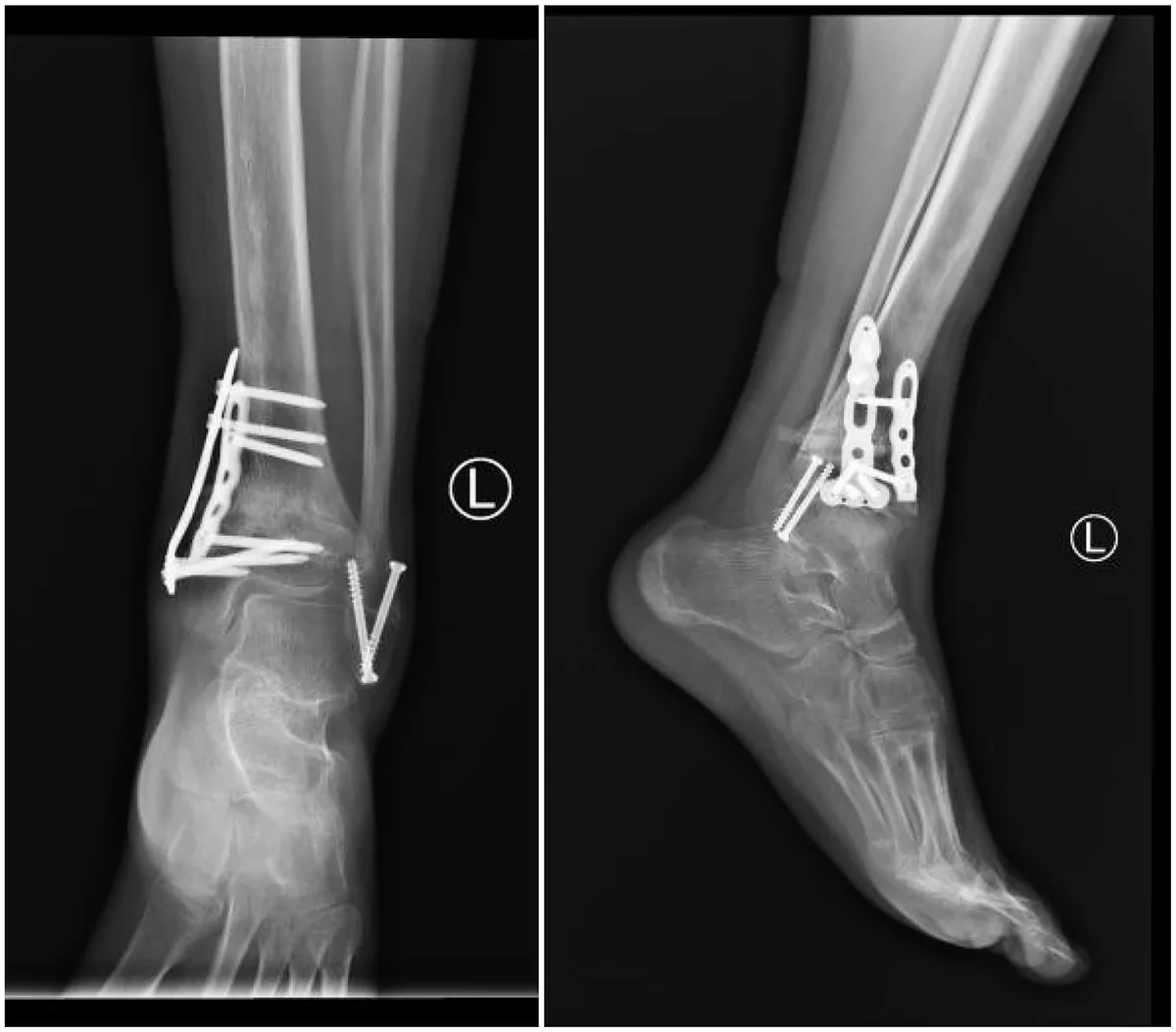
Postoperative radiographs at 8 weeks.

**Figure 7:**
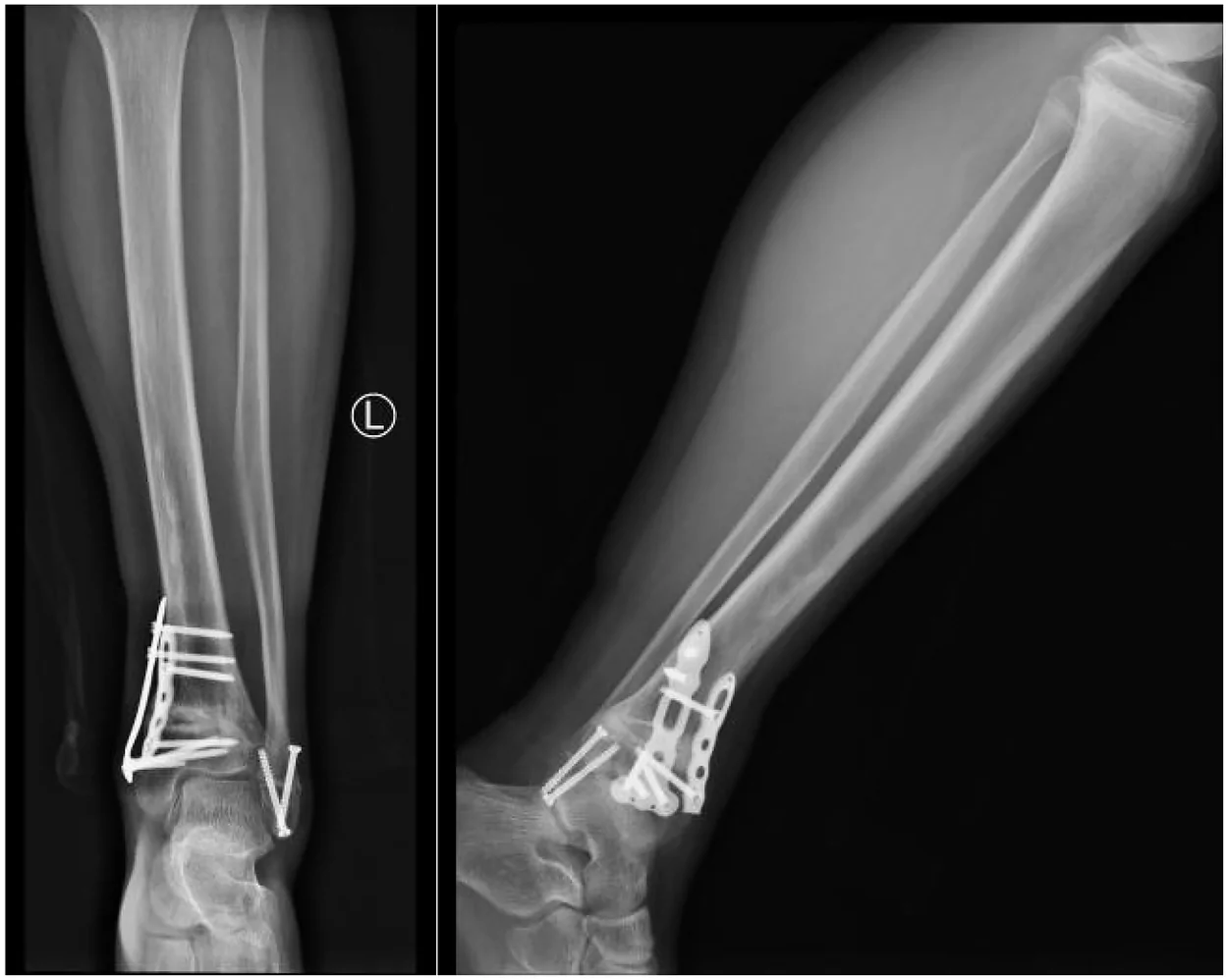
Postoperative radiographs at 6 months.

**Figure 8:**
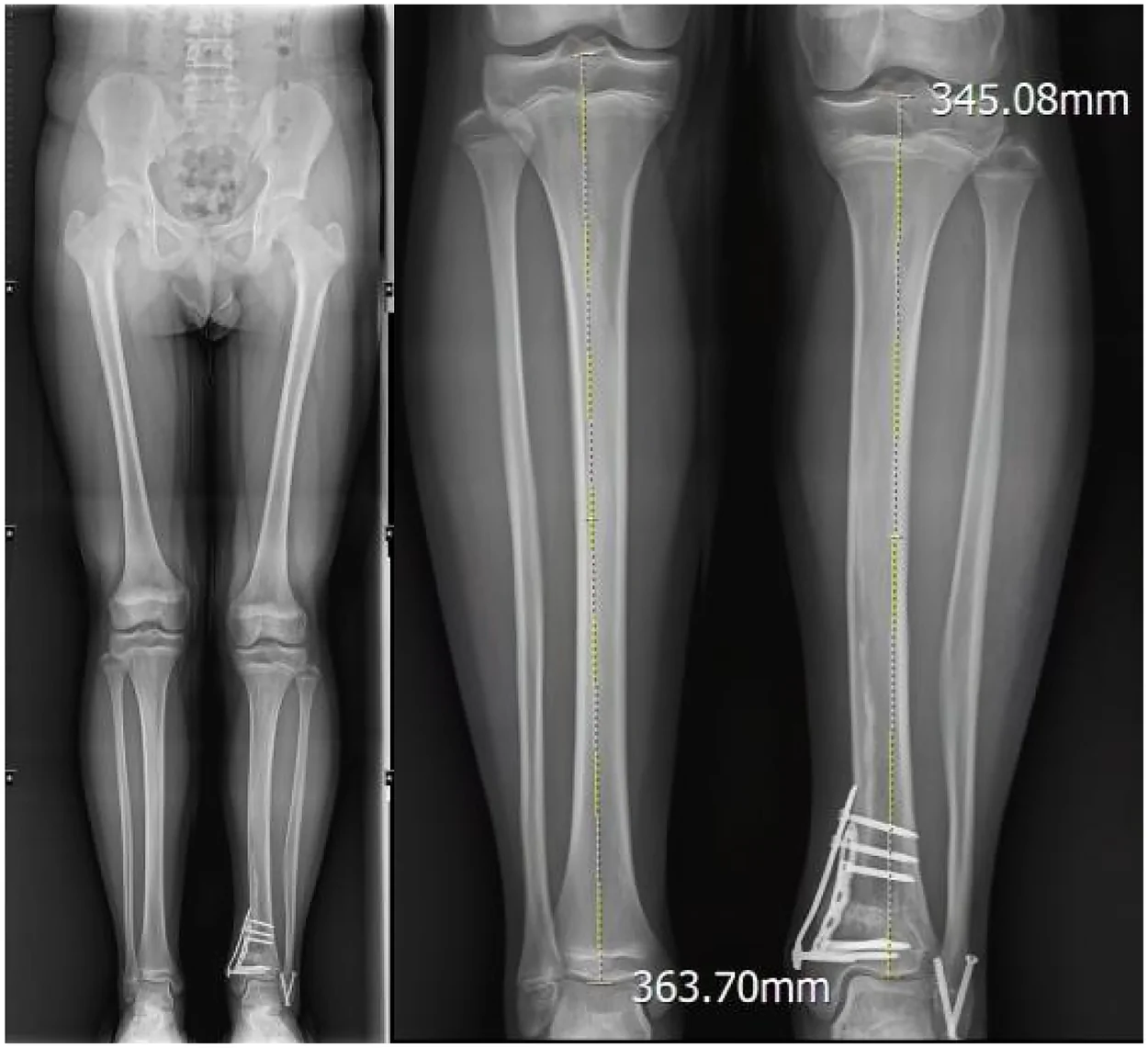
Postoperative radiographs at 12 months.

**Table 1:** Postoperative follow-up data.

Follow-up	Ankle ROM (DF/PF,°)	AOFAS (grade)	VAS (0–10)	Imaging findings	Management
2 wk	2/15	42 (Poor)	3	Not performed	Brace protection; active toe ROM; NWB ambulation
4 wk	8/32	62 (Fair)	0	Early callus at osteotomy; no implant loosening/displacement	D/c brace; start NWB ankle exercises; avoid forceful passive stretch; progress toe WB perception; f/u 4 wk
8 wk	12/38	78 (Good)	0	Continuous callus; partial trabecular bridging; implants *in situ*, intact	Unprotected FWB on level ground (limit to mild discomfort); perimalleolar strengthening; avoid run/jump/deep squat; f/u 3–6 mo
6 mo	15/42	92 (Excellent)	0	Good alignment; osteotomy line obscured by continuous trabeculae (bony union); implants intact	Gradual return to sports; progressive intensity increase; f/u 12 mo
12 mo	18/45	96 (Excellent)	0	TAS ∼90°; cortical union at osteotomy; L tibia physiologically −18 mm vs. R; implants intact	Excellent recovery; elective hardware removal

DF, dorsiflexion; PF, plantarflexion; AOFAS, American Orthopaedic Foot & Ankle Society Ankle-Hindfoot Scale; VAS, visual analogue scale; NWB, non-weight-bearing; FWB, full weight-bearing; WB, weight-bearing; TAS, tibiotalar angle; D/c, discontinue; f/u, follow-up; L, left; R, right; mo, months; wk, weeks.

## Dscussion

The physis is a cartilaginous structure unique to the growing skeleton. Its epiphyseal plate governs longitudinal bone growth and transverse modeling, thereby playing a pivotal role in shaping the final morphology of long bones during childhood and adolescence (9). Physeal infection frequently triggers endochondral ossification within the growth plate, wherein viable cartilage is replaced by pathologic bone, creating a bony bar. Such partial physeal closure acts as a tether, halting longitudinal growth on the affected side and producing progressive angular or torsional deformity (10). Physeal injuries are common in pediatric orthopedics, representing an estimated 15%–30% of all pediatric skeletal injuries (11). Experimental and clinical studies indicate that a physeal bridge occupying more than 7% of the sagittal cross-sectional area portends clinically relevant growth arrest. MRI provides superior soft-tissue contrast and is currently the optimal imaging tool for mapping cartilaginous involvement in the setting of infection (12). The 2021 Pediatric Infectious Diseases Society/Infectious Diseases Society of America (PIDS/IDSA) guidelines state that in children with complicated AHO or cases involving physeal extension, radiographs and magnetic resonance imaging (MRI) should be obtained at the completion of therapy (4). Had the patient in this case undergone serial standing full-length lower-limb radiographs and MRI between 2021 and 2024, the occult physeal injury would have been detected at a still-intervenable stage. Intervening with temporary 8-plate hemiepiphysiodesis during the subclinical phase would have harnessed residual growth to correct alignment gradually, thereby possibly sparing the patient a distal tibial osteotomy altogether (13). Therapeutic strategies for post-infectious distal tibial physeal closure are heterogeneous and lack unified consensus, particularly regarding the timing of osteotomy versus prophylactic epiphysiodesis. These deformities are inherently progressive during growth, carry a risk of recurrence even after successful realignment, and in select cases necessitate revision osteotomy (14). Previous studies suggest that physeal bar resection is a viable option when the extent of physeal involvement is less than 50% and the remaining growth potential is at least 2 years. Conversely, in cases of severe deformity or when insufficient residual growth remains to achieve meaningful correction, corrective osteotomy is indicated (15). Physeal bar resection is most effective in young children with substantial remaining growth; however, complete excision is technically demanding, and the procedure carries an inherent risk of recurrence (16). Preoperative imaging revealed a severe varus deformity with greater than 50% central physeal closure. The remaining growth potential was deemed insufficient to permit spontaneous correction solely through bar resection. An opening-wedge distal tibial osteotomy was favored to avoid iatrogenic limb shortening. Dual-plate fixation—employing a posteromedial T-locking plate and an anteromedial reconstruction plate—yielded superior torsional and axial stability relative to isolated plate or temporary K-wire constructs. This rigid fixation was essential to maintain correction. Interposition of cancellous allograft bone chips into the osteotomy gap provided structural support while promoting osseous union, thereby maintaining the corrected mechanical axis and limb alignment. The rationale for not addressing the bony bridge rested on the understanding that the distal tibial physis accounts for only a small proportion of tibial growth (17). Moreover, with the patient approaching skeletal maturity, the growth reserve was effectively exhausted. Under these conditions, the risk of the residual bony bridge acting as a tether to cause recurrent deformity was considered acceptably low, making aggressive bar resection unnecessary. Preoperatively, the patient exhibited relative fibular overgrowth secondary to tibial growth arrest. While the tibial opening-wedge osteotomy partially corrected this length discrepancy, the distal fibular physis retained growth potential. To preempt further progressive valgus or translational malalignment from ongoing fibular elongation, a concurrent distal fibular epiphysiodesis was performed. In the presence of active infection, external fixation is required to preclude further contamination and minimize the risk of deep infection. However, distal fibular epiphysiodesis arrests lateral ankle growth, necessitating ongoing surveillance for emerging mechanical axis deviations. Should recurrent malalignment become evident, staged secondary intervention may be warranted. Once osseous union is confirmed and the corrected alignment is maintained satisfactorily, elective hardware removal may be performed at the patient's request.

## Patient perspective

The patient reported that although surgical correction has brought great challenges both physically and psychologically. But the subsequent recovery process was very smooth. He was highly satisfied with the treatment results and greatly improved the quality of daily life.

## Conclusion

Although conservative management of AHO may successfully control the acute infection, it can mask occult physeal injuries that subsequently lead to premature physeal arrest and secondary joint deformity. Therefore, the resolution of clinical symptoms should not be regarded as the endpoint of treatment. Regular follow-up is imperative to monitor for latent physeal involvement. In cases where distal tibial physeal arrest has progressed to angular deformity, combined distal tibial opening-wedge osteotomy and simultaneous fibular epiphysiodesis represent an effective and reliable surgical strategy.

## Data Availability

The original contributions presented in the study are included in the article/Supplementary Material, further inquiries can be directed to the corresponding author.
